# Adipose Tissue-Derived Stromal Cells in Matrigel Impact the Regeneration of Severely Damaged Skeletal Muscles

**DOI:** 10.3390/ijms20133313

**Published:** 2019-07-05

**Authors:** Iwona Grabowska, Malgorzata Zimowska, Karolina Maciejewska, Zuzanna Jablonska, Anna Bazga, Michal Ozieblo, Wladyslawa Streminska, Joanna Bem, Edyta Brzoska, Maria A. Ciemerych

**Affiliations:** Department of Cytology, Faculty of Biology, University of Warsaw, Miecznikowa 1, 02-096 Warsaw, Poland

**Keywords:** skeletal muscle, Matrigel, ADSCs, regeneration, TGFβ

## Abstract

In case of large injuries of skeletal muscles the pool of endogenous stem cells, i.e., satellite cells, might be not sufficient to secure proper regeneration. Such failure in reconstruction is often associated with loss of muscle mass and excessive formation of connective tissue. Therapies aiming to improve skeletal muscle regeneration and prevent fibrosis may rely on the transplantation of different types of stem cell. Among such cells are adipose tissue-derived stromal cells (ADSCs) which are relatively easy to isolate, culture, and manipulate. Our study aimed to verify applicability of ADSCs in the therapies of severely injured skeletal muscles. We tested whether 3D structures obtained from Matrigel populated with ADSCs and transplanted to regenerating mouse gastrocnemius muscles could improve the regeneration. In addition, ADSCs used in this study were pretreated with myoblasts-conditioned medium or anti-TGFβ antibody, i.e., the factors modifying their ability to proliferate, migrate, or differentiate. Analyses performed one week after injury allowed us to show the impact of 3D cultured control and pretreated ADSCs at muscle mass and structure, as well as fibrosis development immune response of the injured muscle.

## 1. Introduction

Skeletal muscles are built of the tissue which is characterized by the prominent ability to regenerate after injury. On daily basis many muscles groups are subjected to the damage caused by exercise or accidental mechanical injuries. Next, all of the muscles are affected during aging or in case of degenerative diseases, such as muscular dystrophies. Under physiological conditions skeletal muscle regeneration depends on the muscle specific unipotent stem cells, i.e., satellite cells that remain quiescent residing between sarcolemma and basal lamina surrounding muscle fiber. Injury which destroys muscle fibers, causing the release and/or activation various factors, leads to the satellite cell activation [1]. As a result, these cells start to proliferate, differentiate, and fuse to reconstruct functional muscle fibers. Each time satellite cells become activated to differentiate they also undergo self-renewal, allowing them to sustain their population. Importantly, with aging or disease progression the reservoir of satellite cells might be drained what could lead to the failure in proper skeletal muscle regeneration. Therapeutic approaches to treat such pathologies take into consideration various strategies. Among them is the support of muscle regeneration which could be achieved by the transplantation of cells able to undergo myogenic differentiation. Such cells should be readily available for clinicians, relatively easy to expand in vitro, and able to populate damaged and regenerating tissue. Satellite cells and myoblasts derived from them were among the “obvious” candidates for such cell-based therapies, however, not the perfect ones. The usefulness of these cells was limited by the fact that after transplantation they underwent necrosis, apoptosis, or anoikis [2,3,4,5]; failed to proliferate and colonize either the injured or dystrophic muscle [4,6]; or were eliminated by the host immune system [4,7]. Counteracting these phenomena relied on various experimental interventions, such as manipulating certain signaling pathways enhancing cell survival or migration [8,9,10,11,12,13,14,15] or using various scaffolds and gels to improve cell survival or colonization of injured or dystrophic muscles of mdx mice [16,17,18]. Obstacles preventing the application of satellite cells were among the impulses stimulating the studies on other stem cell types. Importantly, other subpopulations of cells residing within the muscle were shown to be able to undergo myogenic differentiation in vitro and in some cases to participate in and improve muscle regeneration [1]. Among such cells are mesoangioblasts [19,20,21], pericytes [22,23], muscle-resident interstitial cells that do not express paired box protein 7 (*Pax7*) but synthesize cell stress mediator PW1 [24], muscle side population cells [25], or so-called muscle-derived stem cells (MDSCs) [26]. Interestingly, tissue regeneration could be improved by enhancing the colonization of regenerating area by transplanting bio-scaffolds, such as muscle acellular scaffolds [27]. In such cases, transplantation of such extracellular matrix reach biomaterial was shown to attract the resident cells specific for the adjacent tissue.

Except the muscle-residing cells also those ones isolated form other tissues are extensively tested as the ones able to improve skeletal muscle regeneration. Various studies focus at the bone marrow and adipose tissue as the source of so-called mesenchymal stem or stromal cells (MSCs). Bone marrow-derived mesenchymal stromal cells (BM-MSCs) and adipose tissue-derived stromal cells (ADSCs) are characterized by fibroblast-like morphology, ability to adhere and grow in plastic culture dishes, expression of CD73, CD90, CD105 and lack of CD45, CD34, CD14 or CD11b, CD79a, or CD19, and HLA-DR antigens, as well as the ability to undergo at least osteo- and adipogenesis [28,29,30,31]. The fact that ADSC isolation is relatively simple and involves minimally invasive methods causes that these cells are extensively studied. As far as myogenic differentiation of BM-MSCs is concerned they do not possess the ability to undergo this process without additional stimulation. Many lines of evidence prove that MSCs can differentiate into myoblasts and form myotubes either in vitro or in vivo. For example, in vitro cultured BM-MSCs exposed to DNA demethylating agent 5-azacitidine were able to form myoblast-like structures [32]. In vivo, these cells were also shown to be able to incorporate into regenerating injured [33,34] or dystrophic muscles [35,36]. Unfortunately, proportion of myofibers formed with the participation of BM-MSCs was very low, thus, such transplantation did not present sufficient therapeutic potential [37].

As mentioned above, ADSCs could be differentiated, both in vitro and in vivo, to tissues other than adipose one. Multiple lines of evidence document that they could serve as a source for bone cells [38] or even neuronal cells. Santos et al showed that treatment of human ADSCs with cyclic ketamine compounds triggers processes resulting in the changes in the molecular patterns characteristic for neuronal cells [39]. Neuronal differentiation of ADSCs can be also achieved by such different interventions, as manipulation of the level of miRNA-124 [40], overexpression of BNDF [41], or overexpression of Sox2 [42].

In case of ADSCs myogenic potential it was documented both in vitro and in vivo [43]. In fact, some studies suggest that ADSC are more prone to undergo myogenic differentiation than BM-MSCs [44,45,46,47]. Importantly, it is widely accepted that transplantation of these cells, i.e., BM-MSCs and ADSCs, into regenerating muscles often improves regeneration via their immunomodulatory properties [48]. Moreover, such cells could be used in the combination with other factors impacting at various aspects of tissue repair. For example, combination of ADSCs with losartan—transforming growth factor beta (TGFβ) inhibitor—prevented fibrosis and improved repair of regenerating skeletal muscles of mdx mice [49]. TGFβ negatively regulates myoblast proliferation and differentiation [50]. Importantly, it stimulates extracellular matrix (ECM) production, modulates the expression of ECM-degrading enzymes and proteinase inhibitors, resulting in the development of fibrosis in regenerating muscle [51]. Thus, reduction of TGFβ signaling was previously shown by us [52] and others [49,53] to be beneficial for the skeletal muscle regeneration [54].

Next, the cell-based therapy could benefit from the application of various biomaterials or scaffolds mimicking cell niche, securing safe delivery or being a medium to deliver and release beneficial factor. It is also important in case of large, volumetric injures of skeletal muscles, which regeneration might not proceed properly, leading to the excessive connective tissue development. Many lines of evidence document that support of stem cells with materials substituting for extracellular matrix could improve the function and action of transplanted stem cells. For example, as we previously shown, in vitro culture of mouse myoblasts in Matrigel increase the level of adhesion proteins crucial for cell fusion [55]. Moreover, the delivery of cells seeded onto various scaffolds could be the only possible method to deliver stem cells into the site of massive skeletal muscle injury. BM-MSCs transplanted within the Matrigel [56], fibrin [57], or alginate cryogel improved skeletal muscle regeneration [58]. In addition, the latter study involved the application of recombinant growth factors, i.e., IGF-1 and VEGF, to enhance paracrine effect of BM-MSCs [58]. Except the application of selected growth factors the use of the whole secretome present within media conditioned by differentiating myoblasts was also tested as a “tool” to improve regeneration. As previously shown the secretome of differentiating myoblasts contains among other proteins at least 35 growth factors and 40 cytokines [59]. Another study revealed that human differentiating myoblasts secrete at least 253 conventionally, including 43 previously implicated in myogenesis [60]. Importantly, many of these factors were released in extracellular vesicles [60]. Thus, contact with myoblasts or exposure to the myoblast conditioned medium could significantly impact the function of other cells. For example, ADSCs cultured in such conditioned medium proliferated normally, expressed myogenic markers, and presented increased myogenic potential [47]. Moreover, other studies showed that use of media conditioned by differentiating muscles and myofibers had positive impact at regeneration and revascularization of ischemic skeletal muscle [61].

In the current study, using mouse model, we decided to combine various approaches aiming at the improvement of skeletal muscle regeneration—simultaneous application of cells, biomaterial, and growth factors. We choose ADSCs, as cells that could be easily isolated and expanded in combination with Matrigel infused with either anti-TGFβ antibody or myoblast-conditioned medium. We tested if such cell-based approach could positively impact repair of the skeletal muscle large injuries. Thus, as a model we choose mouse skeletal muscles which were induced to regenerate after the volumetric damage.

## 2. Results

The aim of our study was to investigate the impact of mouse ADSCs embedded either in Matrigel infused with medium conditioned by in vitro differentiating C2C12 myoblasts or Matrigel infused with anti-TGFβ antibody. As a control we used Matrigel subjected to control medium or to medium containing TGFβ. We hypothesized that transplantation of Matrigel containing ADSCs and infused with additional factors could be beneficial for the regeneration of muscle which underwent volumetric injury. Matrigel could fill the damaged space and support ADSCs that could impose their immunomodulatory action supported either by growth factors present in conditioned medium or by silencing of TGFβ signaling.

### 2.1. ADSC Reaction to Myoblast-Conditioned Medium or Manipulation of TGFβ Signaling

**Figure 1:**
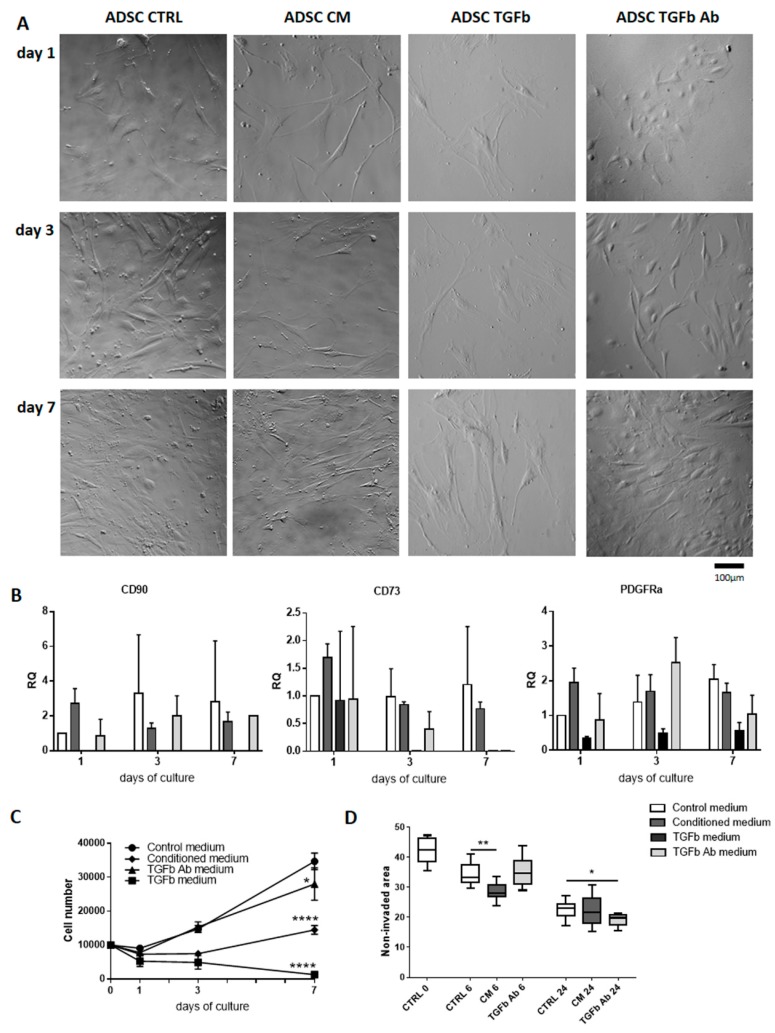
Impact of myoblast-conditioned medium or TGFβ signaling at mouse tissue-derived stromal cells (ADSCs). (**A**) ADSC morphology at 1 (24 h), 3, and 7 day of culture in control (CTRL), myoblast-conditioned (CM), and supplemented either with TGFβ (TGFb) or antibody against TGFβ (TGFb Ab) medium. (**B**) Expression of mRNAs encoding CD90, CD73, and PDGFRα. Expression was related to the levels observed in cells cultured in control medium at day 1, and normalized to the level of mRNA encoding β-actin. RQ: relative quantity. (**C**) ADSC growth curve at 1 (24 h), 3, and 7 day of culture in control, or myoblasts-conditioned, supplemented either with TGFβ or antibody against TGFβ medium. (**D**) In vitro scratch wound healing assay—cells were scratched from culture dish and the area which was not invaded by migrating cells was presented (at 6 h and 24 h). For each time point or experimental group *n* ≥ 3. Data are presented as mean ± SD. *represent results of Student’s *t*-test: * *p* ≤ 0.05; ** *p* ≤ 0.01, **** *p* ≤ 0.0001.

First, we analyzed ADSCs that were cultured in vitro under four different experimental conditions: (1) in control medium, (2) in medium conditioned by differentiating C2C12 myoblasts (at 7th day of culture), (3) medium containing TGFβ, and (4) medium containing antibody against TGFβ. We decided to use TGFβ as a control for the treatment in which signaling dependent on this factor was silenced. ADSC morphology (Figure 1A), expression of mesenchymal cell markers and mesoderm marker (Figure 1B), cell proliferation (Figure 1C), and migration (Figure 1D) was analyzed at day 1, 3, and 7 of culture. Analysis of all types of cultures suggested that conditioned medium influenced the ADSC morphology—cells became more elongated, resembling myoblasts. TGFβ treatment resulted in flattened, outstretched morphology of ADSCs, and significant reduction in their number (Figure 1A). Inhibiting TGFβ signaling with specific antibody did not impacted at cell morphology. Analysis of the expression of mRNAs encoding CD90 (Thy-1) and CD73 (ecto-5’-nukleotydase), which are considered the major markers of MSCs [30], showed their mRNAs were detectable in all types of cell culture at all analyzed stages of cell culture, except the one involving TGFβ. Analysis of mRNA encoding PDGFRα, i.e., the marker of paraxial mesoderm which during embryonic development gives rise to myogenic precursor cells [62], revealed that again its level was the lowest in TGFβ treated cells (Figure 1B). Each of the culture conditions tested decreased the proliferation of ADSCs, however to a different extend (Figure 1C). Thus, TGFβ completely abolished and conditioned medium significantly prevented cell proliferation. Antibody against TGFβ decreased number of cells, as compared to those one cultured in control medium and analyzed at day 7. However, this drop in proliferation was not as profound as in case of conditioned medium. Since TGFβ, used by as a control treatment, completely blocked proliferation of ADSCs it was excluded from subsequent experiments. The influence of culture conditions tested at the migration of ADSCs was assessed using in vitro scratch wound healing assay. The surface of the culture dish from which the cells were removed by scratch (0 h) was calculated. Next, the area which was not invaded by migrating cells was presented. Comparison between control cultures and the other ones showed that at 6 h it was the conditioned medium had a most profound impact at cell migration. At 24 h, however, anti-TGFβ antibody treatment caused the best effects, i.e., the resulted in the biggest area covered by migrating ADSCs (Figure 1D). Thus, the most profound effect at migration could be attributed to anti-TGFβ antibody treatment.

### 2.2. Transplantation of ADSCs Embedded in Matrigel or Matrigel Alone Pretreated with Myoblast-Conditioned Medium or Anti-TGFβ Antibody into Regenerating Muscle

**Figure 2:**
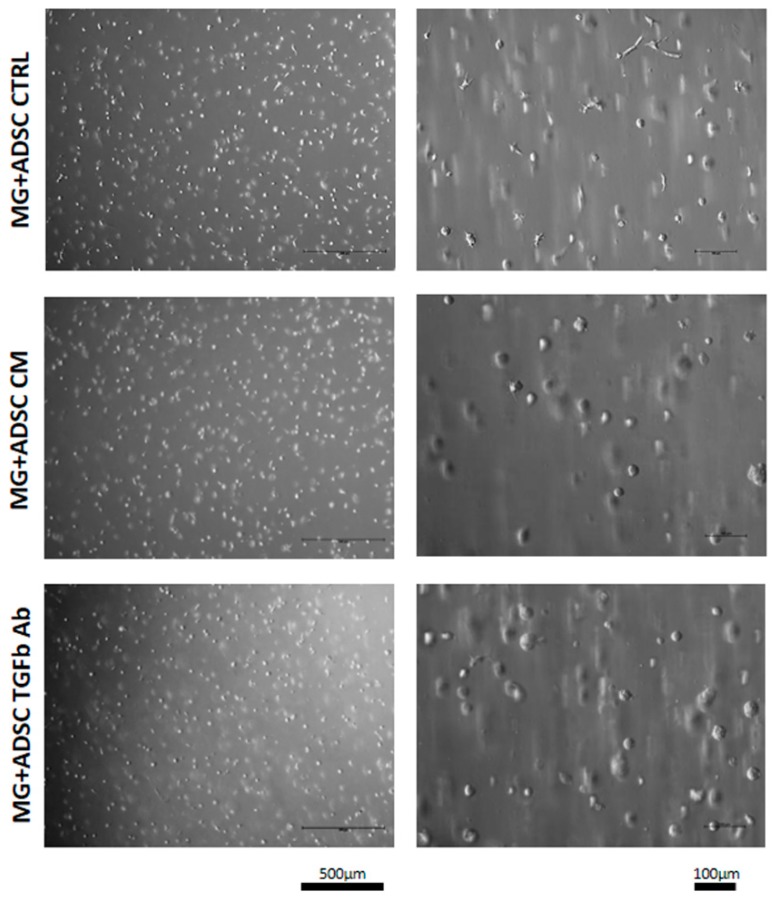
Morphology of ADSCs embedded in Matrigel. ADSC morphology at 48 h of treatment with control (CTRL), myoblast-conditioned (CM), or supplemented with antibody against TGFβ (TGFb Ab) medium.

We showed that ADSC culture in myoblast-conditioned medium or in the presence of anti-TGFβ antibody decreased but not prevented proliferation and have an impact at the migration of these cells. Thus, we decided to test whether ADSCs, supported by Matrigel pretreated with conditioned medium or anti-TGFβ antibody, could improve skeletal muscle regeneration. ADSCs used in this study were labeled by BacMam Transduction Control vector coding GFP what allowed us to visualize position of the cells within the muscle. Matrigel containing ADSCs (7.5 × 10^5^/mL) was preconditioned by incubation with myoblast-conditioned medium or medium containing anti-TGFβ antibody for 48 h. Analysis performed after such pretreatment revealed that cells "suspended" in Matrigel remained round and their morphology was similar regardless of the treatment (Figure 2).

**Figure 3:**
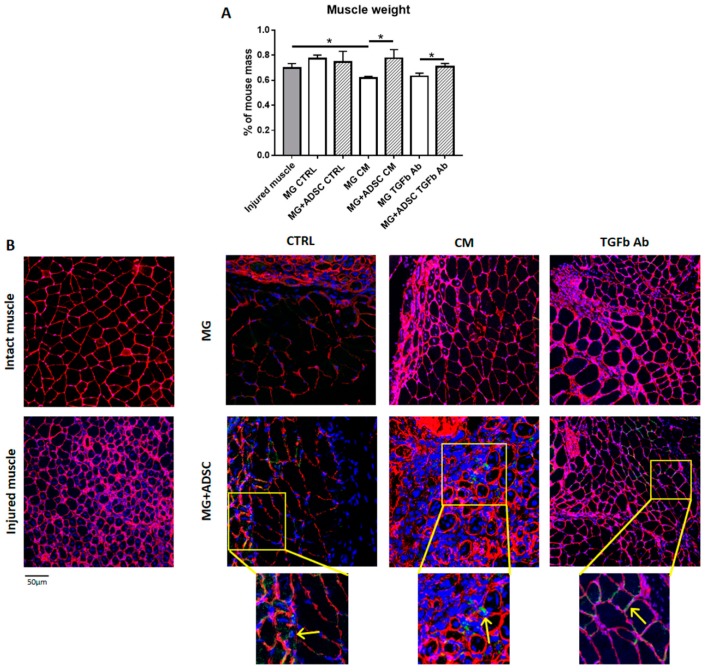
Analysis of skeletal muscles to which ADSCs embedded in Matrigel were transplanted. (**A**) Muscle weight (7 day of regeneration) of injured muscles and muscles that received Matrigel or Matrigel with ADSC pretreated in control (CTRL), myoblast-conditioned (CM), or supplemented with antibody against TGFβ (TGFb Ab) medium. For each experimental group *n* ≥ 3. Data are presented as mean ± SD. * represent results of Student’s *t*-test: * *p* ≤ 0.05. (**B**) Localization of ADSCs in muscles which received Matrigel or Matrigel with ADSC pretreated in control (CTRL), myoblast-conditioned (CM), or supplemented with antibody against TGFβ (TGFb Ab) medium. Inserts: magnification of selected area of muscle cross-sections. Arrows indicates localization of GFP-expressing ADSCs. Green—ADSC-expressing GFP; red—laminin; blue—nuclei. Bar: 50 µm.

**Figure 4:**
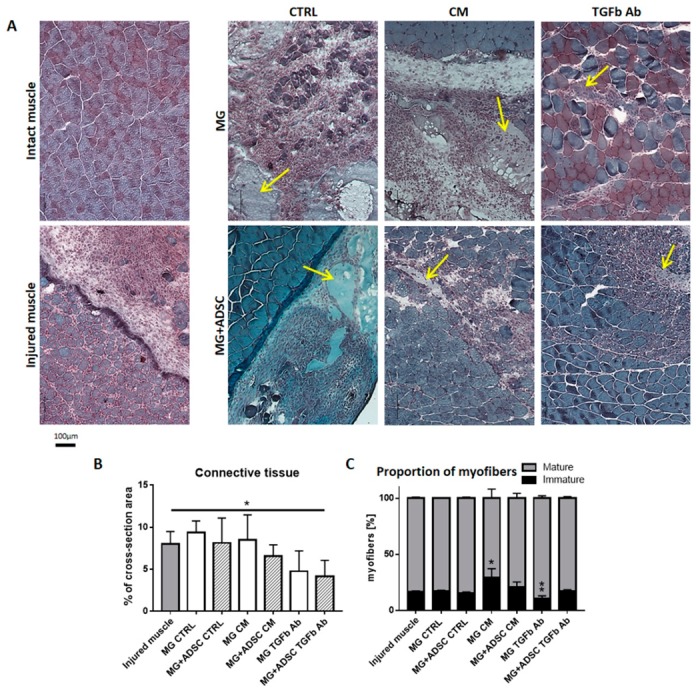
Analysis of skeletal muscle and connective tissue morphology. (**A**) Morphology of skeletal muscles (blue) stained with Harris hematoxylin and Gomori Trichrome dye, at 7 day of regeneration. Intact muscles, injured muscles, and muscles which received Matrigel or Matrigel with ADSC pretreated with control (CTRL), myoblast-conditioned (CM), or supplemented with antibody against TGFβ (TGFb Ab) medium. Arrows indicates localization of Matrigel. (**B**) Area occupied by connective tissue analyzed on cross-sections of injured muscles and muscles which received Matrigel or Matrigel with ADSC pretreated with control (CTRL), myoblast-conditioned (CM) or supplemented with antibody against TGFβ (TGFb Ab) medium. (**C**) Analysis of the proportion of mature and immature muscle fibers present in regenerating skeletal muscles of all analyzed groups. For each experimental group *n* ≥ 3. Data are presented as mean ± SD. * represent results of Student’s *t*-test: * *p* ≤ 0.05, ** *p* ≤ 0.01. Bar - 100 µm.

Matrigel containing ADSCs was then transplanted to gastrocnemius muscle which was injured by deep incision. Transplantation of Matrigel alone or Matrigel containing ADSCs was performed just after injury. Injured muscles or muscles that received Matrigel only served as control. Seven days after transplantation muscles were dissected, weighted (Figure 3A), and processed for further analyzes. Transplantation of ADSCs within the Matrigel which was pretreated with either the myoblast-conditioned medium or anti-TGFβ antibody resulted in higher muscle mass, as compared to muscles that received only Matrigel (Figure 3A). Next, we localized transplanted Matrigel and ADSCs on the basis of GFP fluorescence within the muscle sections in that we also immunolocalized laminin to visualize muscle fiber borders (Figure 3B). Such analysis documented the presence of ADSCs within the muscle tissue. They did not participate in the formation of new myofibers, but were localized between them (Figure 3B). We did not see any substantial differences in ADSC localization between the muscles that received cells within Matrigel treated with control medium, conditioned medium, or medium supplemented with anti-TGFβ antibody. We did, however, notice the differences in the muscle structure. These aspects we analyzed using histological sections (Figure 4A).

We compared histology of intact muscle, and injured muscles at day 7 of regeneration. The transplantation of control or conditioned medium treated Matrigel did not improve muscle regeneration—its structure was comparable to control injured muscles. Thus, the degeneration of injured tissue and accumulation of inflammatory cells was clearly visible. However, the introduction of Matrigel preincubated with anti-TGFβ antibody was beneficial for regenerating tissue—regenerated myofibers were more abundant (Figure 4A). Even better results were achieved when such Matrigel contained ADSCs. In such case improvement of regeneration was noticed in muscles that received Matrigel and ADSCs either conditioned medium treated or exposed to anti-TGFβ antibody (Figure 4A).

Skeletal muscle regeneration is often accompanied with the excessive production of connective tissue fibers. Such adversary effect might hamper proper function of regenerated muscle. Treatments that result in the decrease of connective tissue development and deposition of excessive amounts of extracellular matrix components are beneficial for regeneration [52,63]. Proportion of connective tissue was assessed within regenerating muscles of all group studied, i.e., control ones, transplanted with Matrigel treated with conditioned medium with or without ADSCs, treated with anti-TGFβ antibody containing medium with or without ASCs. Significant reduction of the amount of connective tissue was detected in muscles that received anti-TGFβ antibody pretreated Matrigel-containing ADSCs (Figure 4B).

Analysis of the differences in proportion between mature and immature myofibers, i.e., those ones undergoing reconstruction, showed that the presence of myoblast-conditioned medium treated Matrigel delayed the regeneration, as compared to control injured muscle. In such muscles proportion of myofibers with centrally positioned nuclei was significantly higher, while it was similar in other groups of analyzed muscles (Figure 4B). Interestingly, Matrigel delivering anti-TGFβ antibody significantly accelerated the maturation of muscle fibers, while when it contained ADSCs proportion of immature fibers was again comparable to control, suggesting that cells produced some factors ameliorating the inhibition of TGFβ signaling.

### 2.3. Inflammation-Related Response of Regenerating Muscles

**Figure 5:**
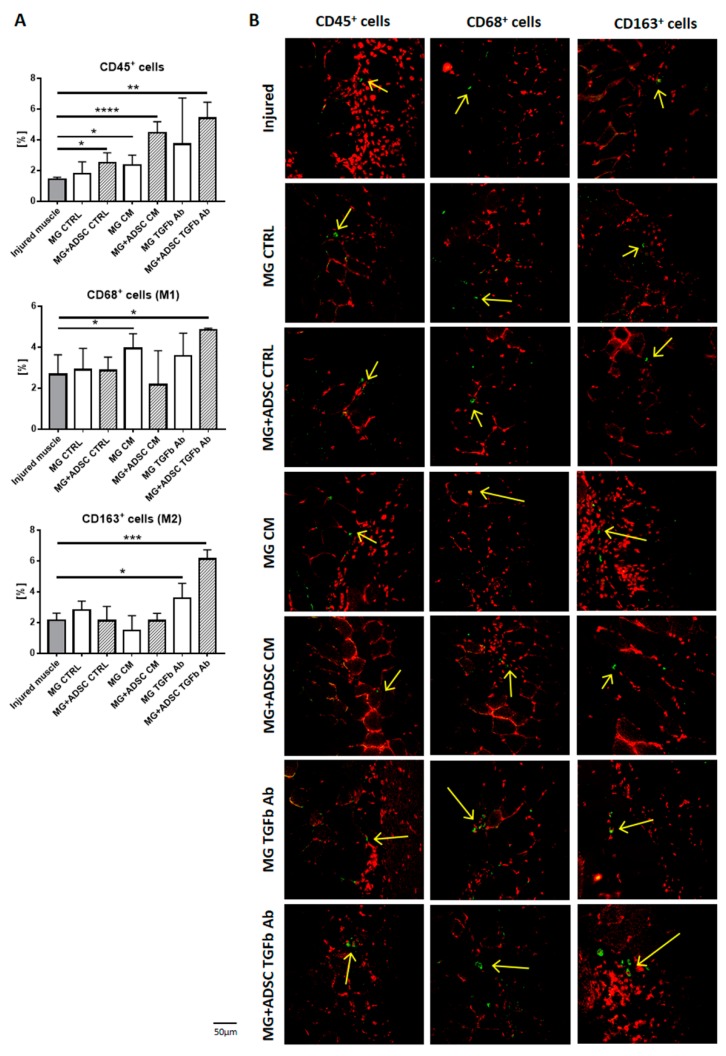
Inflammatory cells in regenerating muscles. (**A**) Proportion of CD45^+^, CD68^+^, and CD163^+^ cells, presented as percentage of all nuclei, present in regenerating skeletal muscles of all analyzed groups. (**B**) Localization of CD45^+^, CD68^+^, and CD163^+^ cells in regenerating skeletal muscles of all analyzed groups. Green - CD45^+^, CD68^+^ or CD163^+^ cells, red - nuclei. Arrows indicates localization of analyzed cells. For each experimental group *n* ≥ 3. Data are presented as mean ± SD. *represent results of Student’s *t*-test: * *p* ≤ 0.05; ** *p* ≤ 0.01, *** *p* ≤ 0.001, **** *p* ≤ 0.0001. Bar: 500 µm.

The successful regeneration of skeletal muscles is a result of properly executed degeneration and regeneration phase. First one is associated with the removal of damaged muscle fibers, infiltration with immune cells and activation of satellite cells. Second one covers the differentiation of satellite cells derived myoblasts into myotubes maturing into myofibers and reconstruction of extracellular matrix. The success of both of the phases depends not only of satellite cells function but to great extent on proper timing of action of immune cells infiltrating the site of injury [64,65,66]. Thus, we analyzed the proportion of lymphocytes characterized by the expression of CD45, proinflammatory M1-macrophages expressing CD68, and anti-inflammatory M2 macrophages expressing CD163 [66,67]. At day 7 after injury the proportion of CD45+ cells was significantly increased in all muscles that received Matrigel with ADSCs, regardless of additional treatments, and also Matrigel pretreated with myoblast-conditioned medium (Figure 5A,B). Next, the influx of proinflammatory M1 macrophages was higher in regenerating muscle implanted with Matrigel pretreated with myoblast-conditioned medium and that one pretreated with anti-TGFβ antibody and containing ADSCs. Importantly, only in latter case, i.e., Matrigel pretreated with anti-TGFβ with ADSCs we noticed significant increase in the proportion of anti-inflammatory M2 macrophages (Figure 5A,B).

**Figure 6:**
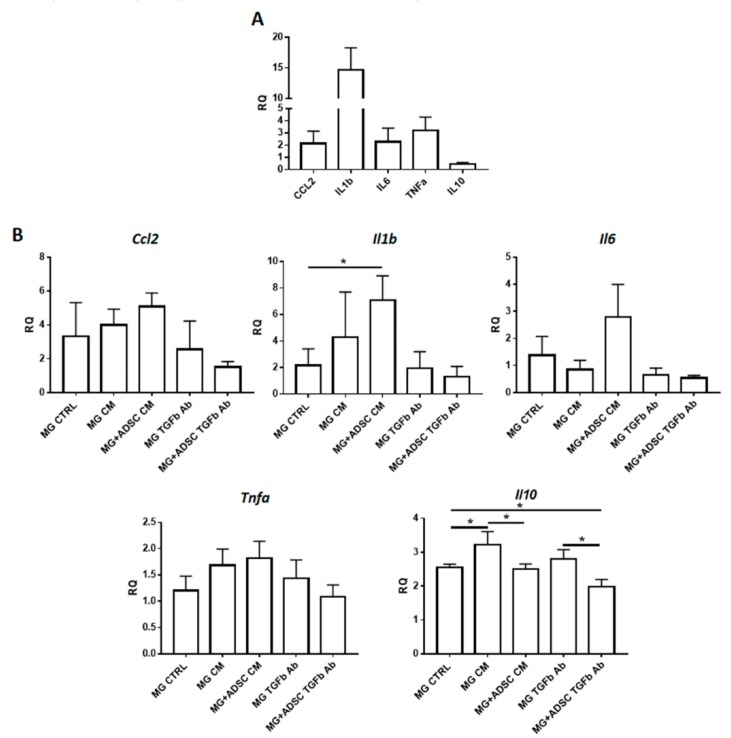
Expression of mRNAs encoding pro- and anti-inflammatory factors. (**A**) Analysis of the level of mRNAs encoding CCL2, IL1b, IL6, TNFα, and IL10 in control injured muscles. (**B**) Expression of cytokines studied in muscles which received Matrigel only or Matrigel with ADSC pretreated in control (CTRL), myoblast-conditioned (CM), or supplemented with antibody against TGFβ (TGFb Ab) medium. Expression was related to the levels observed in injured muscles, and normalized to mRNA encoding β-actin. RQ - relative quantity. For each experimental group *n* ≥ 3. Data are presented as mean ± SD. * represent results of Student’s *t*-test: * *p* ≤ 0.05.

Next, we analyzed the levels of mRNAs encoding selected cytokines playing important role during skeletal muscle regeneration. We have chosen to analyze *CCL2* (C-C Motif Chemokine Ligand 2)—a macrophage-produced cytokine responsible for attracting neutrophils, which is necessary to remove cellular debris [68]; *IL-1b*, *IL-6*, and *TNFα* mediate the inflammatory response and are proinflammatory cytokines produced, e.g., by infiltrated monocytes/macrophages [69,70,71] and *IL-10*—an anti-inflammatory cytokine that regulates changes in macrophage phenotype [72] and improves skeletal muscle regeneration [73,74]. At day 7 after injury the levels of proinflammatory cytokines were increased, as compared to control intact muscle. The level of mRNA encoding anti-inflammatory *IL-10* remained low (Figure 6A). Transplantation of Matrigel alone or Matrigel containing ADSCs, control or treated either with myoblast-conditioned medium or anti-TGFβ antibody did not have substantial beneficial effect at inflammation (Figure 6B). In general proinflammatory cytokines were increased in every analyzed group of muscle, as compared to control injured muscle. Interestingly, muscles that received ADSCs embedded in Matrigel pretreated with anti-TGFβ antibody were characterized by levels of *CCL2*, *IL-1b*, *IL-6*, and *TNFα* similar to that observed in control injured muscle (Figure 6B). *IL-1b* and *IL-6* mRNA levels were increased in muscle transplanted with ADSC containing Matrigel which was pretreated with myoblast-conditioned medium. The mRNA encoding anti-inflammatory IL-10 was upregulated in all analyzed samples, as compared to injured control. Importantly, the presence of ADSCs lowered the expression of these interleukin (Figure 6B).

Thus, an increased proportion of M2 macrophages and level of anti-inflammatory factors in muscles, to which Matrigel pretreated with anti-TGFβ antibody and containing ADSCs was transplanted, significantly improved skeletal muscle regeneration.

## 3. Discussion

Various types of cells are widely tested as a "material" that can be used in therapies supporting tissues and organs repair or function. In such context, the “ideal cell” should present the ability to differentiate into required cell or tissue type and/or support regeneration by other means, e.g., by releasing growth or anti-inflammatory factors. Unfortunately, such ideal stem cell was not identified so far. The best ability to differentiate is attributed to pluripotent stem cells, i.e., embryonic stem cells (ESCs) or induced pluripotent stem cells (iPSCs). They were shown to be extremely easy to propagate and able to differentiate into any given tissue [75,76,77]. In addition, iPSCs could be produced from patient somatic cells what would allow the derivation of syngeneic cells for transplantation. Unfortunately, the methods of pluripotent stem cells differentiation are still not perfect, and some of the cell types, such as skeletal muscle myoblasts, are not easy to derive [37,78]. Moreover, there are also well-founded worries that if not properly prepared these cells can differentiate chaotically within the recipient tissue, forming so-called teratomas, what might pose serious health risk [79]. For these reason multipotent stem cells, such as MSCs and among them ADSCs, attract increasing attention. Their characteristic is quite different from that of ESCs or iPSCs. MSCs could be isolated from various sources, such as bone marrow or adipose tissue, present limited ability to differentiate and for this reason do not form teratomas. However, when appropriately treated they do undergo chondro-, osteo-, and adipogenesis [80,81]. Many lines of evidence indicate that under certain experimental conditions they can also differentiate into such cell types as myoblasts [32] or even those ones of other than mesodermal origin, e.g., neurons [82,83]. However, the efficiency of such differentiation is still not the highest. In vitro analyzes showed that MSCs, isolated from umbilical cord connective tissue are able, for example, to form hybrid myotubes with C2C12 cells [84]. Moreover, MSCs were shown to be able to colonize regenerating muscle and participate in the formation of new myofibers, however, with very low frequency [34,35,36,84]. ADSCs were also documented to be able to undergo myogenic differentiation in vitro or in vivo, i.e., when transplanted to the muscles of *mdx* mice [43]. These and other lines of evidence documented that they are able to colonize the tissue and participate in its regeneration. In our study we introduced Matrigel embedded ADSCs into severely damaged mouse gastrocnemius muscle. Our in vitro study showed that such cells are able to proliferate and migrate under culture conditions applied by us, i.e., either in control, myoblast-conditioned medium, or in the presence of anti-TGFβ antibody. Based on previous reports we also hoped that they will be able to participate in the formation of new myofibers. Unfortunately, ADSCs introduced by us were not able to participate in myofiber regeneration; however, they were detectable within the muscle at day 7 after injury. Such results are in agreement with our previous study during which we were able to detect other MSCs within the regenerating muscle [84,85]. This inconsistency between our findings and those ones of Zhang et al. [43] could be a result of different experimental settings used in these two studies. First of all, we analyzed regeneration of massively injured muscles transplanted with Matrigel containing ADSC which were pretreated either with conditioned medium or anti-TGFβ antibody. Zhang et al studied mdx mouse muscles transplanted with ADSCs that were treated with BIO, bFGF, and forskolin for as long as 7 days, followed by their exposition to ADSC culture supernatant. Such experimental conditions resulted in the participation of these cells in the muscle regeneration. Apparently, the treatment proposed by us is not as efficient. However, despite that ADSCs transplanted within Matrigel did not participate in the skeletal muscle regeneration they were able to impact at this process. Thus, the differentiation into required cell or tissue type is not the action which makes MSCs, such as ADSCs, useful as a tool to improve regeneration. The major impact of MSCs at the regenerating tissue relies at their ability to immunomodulate the function of other cells present within the site of the injury [86,87,88]

We showed that introduction of Matrigel and ADSCs increased the number of CD45+ and CD68+ cells, increasing the local inflammation. However, the most important phenotype we observed in case of CD163+ cells, i.e., anti-inflammatory macrophages which were dramatically increased in skeletal muscles which received anti-TGFβ antibody pretreated Matrigel containing ADSCs. TGFβ modifies activation and proliferation of lymphocytes, induces maturation of monocytes to macrophages, and also acts as macrophage chemoattractant. Thus, the modification of TGFβ level may modulate inflammation and result in the improvement of muscle repair. The development of inflammatory response and its effective silencing are crucial for skeletal muscle regeneration. M2 macrophages play an important role in improving skeletal muscle regeneration. They do not phagocytose degenerating skeletal muscle fibers but rather produce growth factors, such as fibroblast growth factor (FGF) and insulin-like growth factor (IGF-1), and as a result of myoblast proliferation. [73,89,90]. The action of M2 macrophages relies at their secretory activity. Many studies have shown that they secrete various soluble factors as well as release exosomes and by such action may impact at other cell function [91]. Moreover, pretreatment of ADSCs with various growth factors or cytokines may modulate their function [92,93,94,95]. Our current result showed that presence of ADSCs within the transplanted Matrigel significantly improved the structure of regenerating muscle. For example, transplantation of Matrigel treated with myoblast-preconditioned medium resulted in the higher number of immature muscle fibers, as compared to control, i.e., injured muscle. Presence of ADSCs in such Matrigel increased the number of mature fibers, i.e., with peripherally positioned nuclei. Moreover, ADSCs modulated the outcome of anti-TGFβ antibody treatment—in the presence of cells development of connective tissue was significantly limited. Analyzing the level of mRNAs encoding proinflammatory cytokines we noticed that combination of anti-TGFβ antibody infused Matrigel and ADSCs resulted in the level of mRNAs comparable to nontreated, injured muscle. This is especially important since we showed that transplantation of Matrigel increases the level of inflammation, as judged by the expression of *CCL2*, *IL-1b*, or *IL-6*. Thus, the presence of biomaterial might not necessarily be beneficial for the skeletal muscle regeneration and if such is used to improve the regeneration of volumetric injuries it might be of important combine it with certain regeneration-supporting factors or MSCs. In fact, many lines of evidence document that 3D cultures of cells in Matrigel could be beneficial for proliferation and/or differentiation. For example, differentiation of myoblasts cultured in Matrigel was shown to be improved [96]. As far as MSCs are concerned those ones isolated from bone marrow and transplanted within Matrigel was shown to improve soleus muscle regeneration. This was manifested by fewer immature myofibers, however, no impact at fibrosis development was noticed and immune status of such muscles was not analyzed [56].

Combining Matrigel as a stem cell scaffold with additional factors might be another approach to improve tissue repair. The benefits of silencing of TGFβ signaling during skeletal muscle regeneration have been previously shown by us for slow-twitch Soleus muscles, which regeneration is affected by the excessive ECM deposition. Blocking TGFβ action with specific antibodies decreased fibrosis and significantly improved regeneration [52]. Decrease in ECM deposition was also characteristic for muscles treated with such factors inhibiting TGFβ activity as suramin [97], angiotensin receptor blocker [98] or as a result of blocking of TGFβ receptor—TβRI [99]. Importantly, TGFβ was also shown to inhibit proliferation and differentiation of myoblasts [50,100]. Thus, our results documenting complete abolishment of ADSC proliferation by TGFβ and improvement of the regeneration of muscles to which Matrigel pretreated with anti-TGFβ antibody containing ADSCs was transplanted are in agreement with abovementioned findings. The use of myoblast-conditioned medium tested in our study did not appear as beneficial as expected. Transplantation of Matrigel pretreated with such medium resulted with lower muscle mass and affected muscle regeneration, what was documented by increased number of immature myofibers. This effect was counteracted by the addition of ADSCs to such Matrigel. In other experimental setting, however, such medium was shown to improve tissue regeneration impacting revascularization of damaged skeletal muscle tissue [61] or preventing intramuscular adipose tissue differentiation and lipid accumulation [101].

## 4. Materials and Methods

All procedures involving animals were approved by Local Ethics Committee No. 1 in Warsaw, Poland, permissions number 626/2014 (6 December 2014) and 493/2017 (4 January 2018).

### 4.1. Cell Culture

Adipose tissue was isolated from C57BL/6J male mice (aged 6–8 weeks) and transferred to betadine solution (8µL/mL, EGIS Polska sp. z o.o.) and then washed with Phospate Buffered Saline. ADSC were isolated by digestion of fragmented adipose tissue with 0.2% type I collagenase solution (Sigma-Aldrich, Saint Louis, MI, USA) for 90 min at 37 °C. Cells were cultured in Dulbecco’s Modified Eagle’s Medium (DMEM, Invitrogen, Carlsbad, CA, USA) (4.5 g glucose/L) supplemented with 50% FBS (Invitrogen), and gentamycin (Sigma-Aldrich). Cells were cultured at 37 °C in the atmosphere of 5% CO_2_ and analyzed at day 1 (24 h), day 3 and day 7 of culture.

Control ADSCs were cultured under the standard conditions. ADSC were additionally pretreated for 48 h either with 10 μL/mL anti-TGFβ antibody (Santa Cruz Biotechnology, Dallas, TX, USA) or 0.25 ng/mL Recombinant Mouse TGF-β1 (carrier-free) (BioLegend, San Diego, CA, USA) dissolved in 1% Bovine Serum Albumine or conditioned by myoblast medium. Conditioned medium was obtained from confluent culture of fusing/differentiating C2C12 mouse myoblasts cell line. Twenty-four hours before application to ADSC culture, the medium (DMEM 4.5 g glucose/L, supplemented with 15% FBS, and gentamycin was changed in the culture of C2C12 cells. After 24 h the medium was filtered using a 40 μm pore filter. The filtered medium was used for ADSC cell culture. Optimal concentration of reagents and necessary frequency of cell treatments were determined experimentally.

The morphology of cultured cells was analyzed using Nikon Eclipse, TE200 microscope with Hoffman contrast. Cell proliferation was assessed by counting the total number of cells at day 1 (24 h), day 3 and day 7 of culture after their detachment from 10 mm culture dishes using 0.05% trypsin (Invitrogen).

### 4.2. Migration Assay

Migration of ADSC was analyzed using scratch wound healing assay [102]. Briefly, cells were plated in the 8 cm^2^ culture dish and cultured until they reached 90% of confluency. Next, the cells were scratched from the plate using plastic automatic pipette tip to create the “wound.” The wound healing manifested by the ability of the cells to refill the created gap was monitored after 6 and 24 h of culture. Three independent experiments were performed.

### 4.3. qPCR

RNA isolation was performed with the High Pure RNA Isolation Kit (ADSC) or mirVana miRNA Isolation Kit (muscles) according to the manufacturer’s (Roche or Thermo Fisher Scientific, respectively) recommendation. Reverse transcription was performed using 0.5 μg total RNA and RevertAid First Strand cDNA Synthesis Kit (Thermo Fisher Scientific, Waltham, MA, USA), according to the manufacturer’s instruction. qPCR was performed using the following specific TaqMan^®^ probes; mm00493682_g1 (Thy1-CD90), mm00440701_m1 (Pdgfra), mm00501910_m1 (Nt5e-CD73), Mm00441242 (CCL2), Mm00434228 (IL1b), Mm00446190 (IL6), Mm00443258 (TNF-α), and Mm01288386 (IL10), using the TaqMan Gene Expression Master Mix (Thermo Fisher Scientific) and Light Cycler 96 instrument (Roche, Basel, Switzerland). Data was collected and analyzed with Light Cycler 96 SW1.1 software (Roche). Analysis of relative gene expression using quantitative PCR and the 2(T) (-Delta Delta Ct) method was performed according to Livak and Schmittgen [103].

### 4.4. ADSC Labeling Using BacMam GFP Transduction Control

BacMam GFP Transduction Control (Invitrogen) was added to ADSC culture medium (150 μL/12 mL culture medium, i.e., approximately 1.5 × 10^6^ cells). Incubated for 10 min at room temperature and then left overnight in ADSC culture. After this time the BacMam GFP medium was removed and the culture was supplemented with a fresh medium. The procedure was carried out as recommended, based on the manufacturer’s protocol.

### 4.5. Three-Dimensional ADSC Culture in Matrigel and Pretreatment with TGFβ Antibody or Conditioned Medium

ADSC was cultured under standard conditions (monolayer culture) until the desired number of cells was obtained and then cells were labeled with BacMam GFP Transduction Control. Cells marked with GFP marker were washed three times with PBS. Cells were removed from the dish by adding trypsin and a few minutes incubation (~5–7 min) at 37 °C. Suspended cells were transferred to a centrifuge tube and centrifuged at 1300 rpm for 8 min. After centrifugation the supernatant was discarded and the sediment was suspended in ADSC culture medium to obtain solution of 250,000 in 10 μL of the medium. The next steps in the procedure were carried out on ice, i.e., 290 μL of Matrigel was collected and then 10 μL of cell suspension was added (250,000 ADSC). The cells in Matrigel were distributed by pipetting several times and the suspension was transferred to the 1 cm^2^ well of the 4-well dish. In variants without cells 300 μL of Matrigel was collected and transferred to the dish. The procedure was repeated until all the necessary variants were prepared. The Matrigel was maintained at a temperature of about 4 °C all the time in order to keep it in the liquid form. Then the Matrigel seeded with cells or Matrigel itself was incubated for 30 min at 37 °C to form a gel, after which 300 μL of medium (control, or containing 10 μL/mL anti-TGFβ antibody, or 0.25 ng/mL Recombinant Mouse TGF-β1, or conditioned by myoblasts medium) was added to each well. Matrigel with cells was photographed (Nikon TE200 microscope using NIS Elements program, Minato, Tokyo, Japan). The 3D structures were left in the incubator (37 °C, 5% CO_2_) for 48 h. After this time the medium was discarded and the gel transplanted into the muscles.

### 4.6. Skeletal Muscle Injury and Transplantation of Matrigel

After the animals (6–8-week-old male C57BL/6J) were anesthetized with isoflurane the surgical field, was cleared from fur and this area was topically anaesthetized with 4% Lidocain. Next, right gastrocnemius muscle was exposed (from the Achilles tendon to the knee) by cutting the skin and making 3-mm-long and 3-mm-deep incision within the middle part of the muscle belly. Then Matrigel with/without ADSC cells was introduced to such site of muscle injury and the skin was sutured. Each variant of the experiment was performed in three biological repeats. After the procedure mice were kept under standard conditions with free access to food and water. Additional control was provided by (1) muscles not subjected to any procedures (intact) and (2) muscles injured (without ADSC transplantation in Matrigel)—three biological repeats for each variant. After 7 days mice were killed by spinal cord dislocation. Muscles and mouse from which the muscle was taken were weighed. The isolated muscles were frozen in isopentane cooled in liquid nitrogen and stored at −80 °C.

### 4.7. Histological Analyzes—Myofibers Number and Connective Tissue Area

The frozen muscles were cut into sections of 10 μm thickness using cryostat (Microm HM505N). Cross-sections were placed on slides and after drying were stored at 4 °C. Sections were hydrated in PBS (10 min), and then stained in Harris hematoxylin solution for 40 min, washed gently under tap water (about 5 min), and stained in Gomori trichrome solution (30 min). After staining, sections were rinsed again under tap water (~5 min). Dehydrated in 96% ethanol (2 × 3 min) and then in 100% ethanol (2 × 3 min). Dehydrated preparations were immersed twice in Neoclear xylene equivalent (2 × 8 min), and closed with Entalan. The samples were analyzed using Nikon TE200 microscope and NIS Elements program. The number of newly formed myofibers (immature) was determined in relation to the number of undamaged myofibers (mature) at the day 7 of regeneration. The photos of sections from each of the 3 replicates for each variant were analyzed and the results were presented as a proportion of the number of mature and immature myofibers on the sections. The area occupied by the connective tissue in relation to the area of the entire section was calculated using ImageJ software. The sections of each of the 3 repetitions for each variant were analyzed. The obtained data were averaged and presented on a graph.

### 4.8. Immunolocalization

In the first stage muscle cross-sections were rehydrated for 10 min. in PBS, and then fixed for 10 min in 3% paraformaldehyde (Sigma-Aldrich) in PBS. Next, cells or muscle sections were permeabilized with 0.1% Triton X-100/PBS (Sigma-Aldrich) and incubated with 0.25% glycine for 15 min (Sigma-Aldrich). Nonspecific binding of antibodies was blocked with 3% bovine serum albumin (BSA, Sigma-Aldrich) in PBS, at room temperature, for 30 min. Next, sections were incubated for with primary antibodies: rabbit against Laminin (Sigma-Aldrich), rat against CD45 (Abcam), rat anti-CD68 (Abcam), and rabbit anti-CD163 (Abcam) diluted 1:100 in 3% BSA in PBS overnight, washed with PBS, and incubated at room temperature with secondary donkey antibodies with Alexa Fluor 594, (Life Technologies, Carlsbad, CA, USA) diluted 1:200 in 3% BSA in PBS. After washing with PBS, cell nuclei were visualized by incubation with DRAQ5 (Biostatus Limited, Shepshed, Loughborough, Great Britain) diluted 1:1000 in PBS for 10 min. Specimens were mounted with Fluorescent Mounting Medium (Dako Cytomation, Glostrup, Denmark). After the procedure was completed samples were analyzed using Axio Observer Z1 scanning confocal microscope (Zeiss, Oberkochen, Germany) equipped with LSM 700 software (Zeiss). For the analysis of leukocytes and macrophages presence the photos of sections from each of the 3 independent replicates for each variant were analyzed and the results were presented as a proportion of the number of CD45^+^, CD68^+^, or CD163^+^ cells and all nuclei on the 3 pictures of each of three sections were analyzed. The obtained data were averaged and presented on a graph.

### 4.9. Statistical Analysis

Results were analyzed using GraphPad Software (San Diego, CaliphCA, USA) and nonpaired *t*-test was performed to compare treated with the control cells/muscles. The differences were considered statistically significant when *p* < 0.05. Each analysis was repeated three times. Data are expressed as mean ± standard deviation. Statistical significance was determined using a Student’s *t*-test - * *p* < 0.05; ** *p* < 0.01; *** *p* < 0.005.

## 5. Conclusions

ADSCs can be considered as a suitable material for replacement therapies. Results of our study document that transplantation of preconditioned ADSCs in Matrigel 3D structures in combination with silencing of TGFβ signaling could improve skeletal muscle regeneration as judged by muscle mass and structure, proportion of mature myofibers, decreased level of fibrosis, and an increase in the number of anti-inflammatory macrophages and appropriate levels of inflammation-regulating factors. Thus, by silencing this signaling pathway the regeneration could be improved. However, the molecular basis of this phenomenon is still unclear. Elucidation of molecular changes triggered by silencing TGFβ signaling would uncover more precise targets which could be “used” as a tool to improve skeletal muscle regeneration. Also other preconditioning treatments, such as application of selected cytokines, in combination with 3D scaffolds should be investigated.

## Abbreviations

ADSC	adipose tissue-derived stromal cell
BM-MSC	bone marrow-derived mesenchymal stromal cell
CM	myoblast-conditioned medium
CTRL	control medium
ECM	extracellular matrix
ESC	embryonic stem cell
FBS	fetal bovine serum
iPSC	induced pluripotent stem cell
MDSC	muscle-derived stem cell
MG	Matrigel
MSC	mesenchymal stem/stromal cell
TGFβ	transforming growth factor beta
TGFb	medium supplemented with TGFβ
TGFb Ab	medium supplemented with antibody against TGFβ
